# Navigating the Parasitic Landscape: Insights into Infection Patterns and Public Health Strategies in West Africa

**DOI:** 10.3390/tropicalmed10050125

**Published:** 2025-05-06

**Authors:** Patrick F. Ayeh-Kumi, Patience B. Tetteh-Quarcoo, Isabella N. A. Aryee, Peter Nii Apai Baddoo, James Teye Ocansey, Michael Kofi Otoboah

**Affiliations:** Department of Medical Microbiology, University of Ghana Medical School, Korle-Bu, Accra P.O. Box KB 4236, Ghana; isabellaaryee2001@gmail.com (I.N.A.A.); peterbaddoo19@gmail.com (P.N.A.B.); ocanseyjamesteye@gmail.com (J.T.O.); michaelotoboah@gmail.com (M.K.O.)

**Keywords:** parasitic infections, transmission, zoonotic disease, mortality, diagnostic disease

## Abstract

Parasitic infections remain a significant public health challenge in West Africa, contributing to high morbidity and mortality rates, economic burdens, and healthcare system strain. Malaria, soil-transmitted helminths, schistosomiasis, and other parasitic diseases persist due to environmental, socio-economic, and healthcare barriers. A systematic literature search was conducted using databases such as PubMed, Scopus, Web of Science, and Science Direct. Studies published between 2014 and 2024 were screened using predefined eligibility criteria. Cross-sectional and case–control studies reporting on prevalence, diagnostic techniques, and treatment of parasitic infections in West Africa were included. The Rayyan online platform was used for screening, and data extraction focused on study location, prevalence rates, diagnostic methods, and treatment regimens. This review identified the high prevalence rates of malaria, schistosomiasis, and soil-transmitted helminths across various West African countries. Co-infections were frequently reported, particularly among children and pregnant women. Diagnostic methods ranged from traditional microscopy to advanced molecular techniques, though accessibility remained a challenge in resource-limited settings. Treatment strategies, including artemisinin-based combination therapies for malaria and mass drug administration for helminths, showed varying efficacy due to reinfection risks and emerging drug resistance. Factors influencing transmission included environmental conditions, sanitation practices, socio-economic status, and healthcare access. Parasitic infections in West Africa continue to pose significant health and economic challenges. Integrated control programs, enhanced surveillance systems, improved access to diagnostics and treatment, and targeted public health interventions are essential for reducing disease burden. Further research is needed to evaluate the long-term impact of existing interventions and explore innovative solutions for parasite control and elimination.

## 1. Introduction

Parasitic infections in West Africa are not just a health issue: they are silent thieves, stealing an estimated 12 billion dollars annually from the continent’s economy due to lost productivity and healthcare costs, while millions suffer in silence. Parasites are organisms that live on or within a host, deriving nutrients at the host’s expense, a relationship known as parasitism. This type of symbiosis is characterized by the parasite benefiting while the host suffers harm [1]. The diversity of parasites affecting humans in West Africa is striking, with various types including protozoa, helminths, and ectoparasites, each posing unique challenges to public health. For instance, protozoan infections such as malaria, caused by *Plasmodium* spp., remain a leading cause of morbidity and mortality in the region, particularly among children and pregnant women [2]. Schistosomiasis, resulting from *Schistosoma* spp., is endemic in many freshwater areas and affects millions of individuals, often leading to chronic health issues [3]. Lymphatic filariasis, caused by Wuchereria bancrofti, is another significant concern, resulting in severe disfigurement and disability that can affect social and economic well-being [4]. Additionally, soil-transmitted helminthiases (STH) caused by Ascaris lumbricoides, Trichuris trichiura, and hookworms are prevalent due to inadequate sanitation and hygiene practices [5]. Amoebic dysentery, resulting from Entamoeba histolytica infection, further complicates the health landscape by causing severe gastrointestinal symptoms [6]. The interplay of these parasitic infections highlights the urgent need for effective public health strategies to combat their prevalence and mitigate their impact on human health in West Africa.

### 1.1. Prevalence

The prevalence of parasitic infections in West Africa is alarmingly high, significantly influencing public health and socio-economic stability. Malaria remains the most prevalent parasitic disease, with the World Health Organization estimating that there were approximately 241 million cases globally in 2020, with a substantial proportion occurring in West Africa, particularly in countries like Nigeria, Ghana, and Côte d’Ivoire [2,7]. Schistosomiasis affects over 200 million people worldwide, with West Africa accounting for a significant number of cases; Nigeria alone has one of the highest burdens, with an estimated 29 million individuals infected [8]. In addition, a study in Mpraeso, Ghana reported that children were at higher risk of asymptomatic parasitemia making them potential reservoirs. This raises concern about the spread of infection by the asymptomatic patients consequently leading to higher prevalence in the region [9]. Lymphatic filariasis also poses a serious threat, with about 65 million people at risk in the region, particularly in Nigeria and Ghana. STH infections affect approximately 40% of children in endemic areas, translating to millions of affected individuals across countries like Benin and Togo [10].

In West Africa, intestinal parasitic infections (IPIs) are prevalent among various demographic groups. For instance, research in the Democratic Republic of São Tomé and Príncipe reported a staggering prevalence of 64.7% among school children, underscoring the widespread nature of these infections despite ongoing deworming programs [11]. Furthermore, these diseases disproportionately affect vulnerable populations, including children under five years and pregnant women. A study in Accra, Ghana, found a 24% prevalence of malaria among febrile children, with significant associations between malaria and anemia (72% prevalence) [12]. The high prevalence rates underscore the urgent need for targeted public health interventions to address these parasitic infections in West Africa. The economic implications of these infections are substantial. Parasitic diseases have led to decreased productivity due to increased absenteeism from work and school, as well as long-term health complications that reduce an individual’s ability to contribute economically. For instance, the World Bank estimates that malaria alone costs African economies approximately USD 12 billion annually in lost productivity [13].

STH remain a significant public health concern in West Africa, with varying prevalence rates across the region. Recent studies indicate a substantial reduction in STH prevalence among children aged 5–14 years, decreasing from 44% in 2000 to 13% in 2018 due to improved preventive measures and sanitation efforts [14]. However, certain areas still report high infection rates; for instance, 59% of children aged 6–13 years in the Brong Ahafo region of Ghana were found to be infected with *hookworm* [15]. A pooled analysis of 57 studies involving 49,630 school-aged children across various regions revealed an overall STH infection prevalence of 37.16%, with specific rates for different species: *Ascaris lumbricoides* at 24.07%, *Trichuris trichiura* at 16.04%, and *hookworm* at 10.20% [16]. Despite ongoing control programs, STH infections continue to pose challenges, especially in areas where sanitation remains inadequate, highlighting the need for sustained intervention efforts to further reduce these infections and their associated health impacts.

Furthermore, the burden of healthcare costs associated with treating these infections can strain public health systems and divert resources from other critical areas of development [17]. In terms of social development, parasitic infections can perpetuate cycles of poverty and inequality. Communities heavily affected by these diseases often experience reduced educational attainment due to absenteeism and cognitive impairment caused by infections like *schistosomiasis*, which can affect children’s learning abilities [18]. High mortality rates are observed in infections such as malaria and visceral *leishmaniasis*, which have devastated populations and disrupted social structures.

### 1.2. Factors Influencing Parasitic Disease Transmission

Parasitic disease transmission refers to the process through which parasites are transferred from one host to another, allowing them to infect and derive nutrients at the host’s expense. For illustration, in West Africa, variations in altitude, climate, and local mosquito populations significantly affect the transmission rates of *Plasmodium falciparum*, the most prevalent malaria-causing parasite. A complex interplay of environmental factors, human behavior, and socio-cultural dynamics has influenced the transmission of parasitic infections in West Africa. Environmental factors play a critical role in the lifecycle of many parasites, particularly those that rely on specific ecological conditions for their transmission. For instance, the presence of stagnant water bodies is essential for the breeding of mosquito vectors that transmit malaria and other diseases. Similarly, freshwater sources are critical for the lifecycle of *Schistosoma* spp., which requires specific snail hosts to complete their development [19]. Climate change and seasonal variations have also affected the distribution and abundance of these vectors, leading to fluctuations in infection rates. Human behavior has significantly influenced parasite transmission as well. Practices such as inadequate sanitation, poor hygiene, and unsafe water usage can facilitate the spread of infections like *schistosomiasis* and *soil-transmitted helminthiases*. In many communities, cultural beliefs and practices surrounding health and illness can affect an individual’s willingness to seek treatment or adhere to preventive measures. For example, traditional medicine is preferred over conventional treatments, leading to delays in seeking effective care [20]. Furthermore, socio-economic factors such as poverty and education level are closely linked to the prevalence of parasitic infections. Populations with limited access to healthcare services are often more vulnerable to infections due to a lack of resources for prevention and treatment. Disparities in health education have also resulted in varying levels of awareness about transmission routes and preventive strategies among different communities [21].

In West Africa, agricultural practices are deeply intertwined with the transmission dynamics of parasitic infections, as many communities depend on farming for their livelihood. The expansion of irrigated agriculture in countries like Mali, Burkina Faso, and Nigeria has been pivotal for food security but has also facilitated the transmission of waterborne and vector-borne parasitic diseases [22]. For example, rice paddies and irrigation canals provide breeding grounds for snails, the intermediate hosts of *Schistosoma* spp., leading to an increased prevalence of schistosomiasis in farming communities. A study conducted in Mali found that schistosomiasis prevalence among nearby populations was significantly higher compared to non-irrigated areas, underscoring the health impact of large-scale irrigation projects [23]. The use of untreated animal manure and human waste as fertilizer—a practice prevalent in smallholder farming systems in countries like Ghana and Senegal—may have also contributed to the spread of soil-transmitted helminths. These parasites, including *Ascaris lumbricoides* and *Trichuris trichiura*, contaminate the soil and crops, creating pathways for human infection [16]. One study in Ghana revealed that farmers using partially treated and untreated water had significantly higher rates of helminth infections [24].

Livestock farming is another key factor in the region. The traditional practice of open grazing, common in countries such as Nigeria, Côte d’Ivoire, and Togo, brought humans into close contact with cattle, goats, and other livestock that may carry zoonotic parasites. A study on *Cryptosporidium* focused in Africa, suggested that although there could be some species and subtypes of cryptosporidium affecting both animal and human health, there is a need for in-depth studies to establish the animal-human (zoonotic) transmission [25]. In addition, the trade and slaughter of livestock without proper veterinary oversight contribute to the transmission of *Echinococcus granulosus*, causing hydatid disease in humans, particularly in rural areas. Furthermore, deforestation for agricultural expansion is another driver of parasitic infection spread in West Africa. Clearing forests for cocoa, palm oil, and cassava plantations in Côte d’Ivoire, Liberia, and Ghana has altered ecosystems and increased human exposure to parasitic vectors. For example, deforestation has been linked to the rise in cutaneous leishmaniasis cases, as humans encroach on habitats previously dominated by Leishmania vectors [26].

### 1.3. Morbidity and Mortality

Parasitic infections significantly affect human health in West Africa, contributing to high morbidity and mortality rates. The mortality associated with malaria remains a critical public health issue, particularly in Africa, where the majority of deaths occur. In 2020, malaria accounted for approximately 627,000 deaths globally, with a significant concentration in West Africa, highlighting the urgent need for effective interventions. Africa reported 95% of malaria cases and 96% of deaths, indicating a severe burden on the continent. Western Sub-Saharan Africa is the only region where malaria deaths increased from 1990 to 2019, emphasizing its ongoing crisis [27,28]. *Schistosomiasis* and *lymphatic filariasis* also contributed to high morbidity rates, causing chronic health issues such as organ damage and severe disability, which can hinder daily functioning and quality of life (Rahim and Karim, 2022) [10].

West Africa faces significant challenges in morbidity and mortality, particularly regarding child and maternal health. The region has the highest under-five mortality rate (U5MR) in sub-Saharan Africa, with approximately 95.3 deaths per 1000 live births as of 2019, compounded by inadequate healthcare access, poor nutrition, and prevalent infectious diseases [29]. Neonatal mortality rates are also alarming at around 30.5 deaths per 1000 live births, with neonatal conditions being a leading cause of death among children under five. The maternal mortality ratio (MMR) is critically high at 724 deaths per 100,000 live births, with some countries like Chad and Nigeria exceeding 1000 per 100,000 [30]. STH further contributed to morbidity, particularly among children; for example, hookworm infections can affect up to 59% of children aged 6–13 years in certain regions of Ghana. Although mass drug administration programs have been implemented to combat STHs, challenges remain due to sporadic coverage and varying prevalence across different areas [16].

### 1.4. Diagnostics and Treatments

The field of parasitology has evolved significantly in recent years, with a variety of diagnostic techniques being employed to detect and identify parasitic infections. Traditional methods, such as microscopy, remain foundational for diagnosing many parasitic diseases. Microscopic examination of stool samples, blood smears, and tissue biopsies allows for the direct visualization of parasites, with techniques like the Giemsa stain being standard for malaria diagnosis. However, these methods are labor-intensive and require skilled technicians, leading to limitations in sensitivity and specificity, particularly for low-parasite-load infections [31]. To address these challenges, molecular techniques have gained prominence. Polymerase Chain Reaction (PCR) and its variants, such as loop-mediated isothermal amplification (LAMP), offer high sensitivity and specificity for detecting parasite DNA or RNA, enabling the identification of species even in complex samples [32]. For example, PCR has been shown to have a diagnostic accuracy of up to 95% for certain parasites [33]. Additionally, serological tests like enzyme-linked immunosorbent assays (ELISA) are widely used to detect specific antibodies or antigens related to parasitic infections, providing valuable information when direct detection methods are inconclusive [34]. Recent advancements also include innovative approaches such as DNA barcoding and artificial intelligence (AI)-based image analysis. DNA barcoding allows for precise species identification through genetic sequencing, achieving accuracy rates around 95% [33]. AI technologies are being developed to analyze images of parasites and automate identification processes, which could significantly reduce diagnostic turnaround times. Despite these advancements, challenges remain in the form of high costs associated with some molecular techniques and the need for specialized equipment and training.

Current treatment options for parasitic infections in West Africa vary depending on the specific pathogen involved, with several effective therapies available for the most common infections. For malaria, the primary treatment is artemisinin-based combination therapies (ACTs), which have been shown to be highly effective against *Plasmodium falciparum*, the deadliest malaria parasite. However, the emergence of drug-resistant strains in some regions poses a significant challenge to malaria control efforts [35]. Schistosomiasis is typically treated with praziquantel, which is effective against all species of Schistosoma and has a high cure rate. While praziquantel is generally, well-tolerated and effective, limitations arise from its inability to prevent reinfection and its ineffectiveness against juvenile worms [36]. For soil-transmitted helminth infections, Albendazole and Mebendazole are commonly used, but their efficacy can be compromised by poor sanitation and reinfection rates [37]. In addition to these treatments, amoebic dysentery, caused by *Entamoeba histolytica*, is treated with metronidazole or tinidazole, which are effective against the trophozoite form of the parasite. However, treatment efficacy can be limited by the presence of asymptomatic carriers who can continue to spread the infection [38]. Visceral leishmaniasis, another significant parasitic disease in some West African regions, is treated with liposomal amphotericin B or miltefosine. While these treatments are effective, their availability can be limited due to high costs and logistical challenges in rural areas [39]. Human African trypanosomiasis (HAT), also known as sleeping sickness, is treated based on the causative subspecies (*Trypanosoma brucei gambiense* or *Trypanosoma brucei rhodesiense*) and the disease stage. For *T. b. gambiense* (TbG), prevalent in West Africa, early-stage treatment traditionally relied on pentamidine, administered intramuscularly or intravenously, which is effective but associated with side effects such as hypotension and hypoglycemia. Late-stage TbG HAT, involving central nervous system invasion, historically required melarsoprol. Eflornithine later emerged as a safer alternative for late-stage TbG but necessitated complex intravenous administration. The introduction of fexinidazole by the World Health Organization (WHO) in 2024 revolutionized HAT treatment by providing an all-oral regimen effective for both early and late stages of TbG HAT [40]. Fexinidazole simplifies treatment in resource-limited settings by eliminating the need for hospitalization and intravenous administration, making it accessible for patients aged six years and older who weigh at least 20 kg. Additionally, nifurtimox-eflornithine combination therapy (NECT) remains an alternative for late-stage TbG cases where fexinidazole is unsuitable [40] (WHO, 2024). The efficacy of these treatments varies widely based on factors such as the stage of infection, patient adherence to treatment regimens, and the presence of co-infections. For instance, while ACTs for malaria are highly effective when administered correctly, issues such as patient non-compliance and incomplete treatment courses can lead to treatment failures and contribute to resistance [41]. Similarly, praziquantel’s effectiveness against schistosomiasis is compromised by reinfection due to ongoing exposure in endemic areas [42].

A growing concern across all these treatments is the issue of drug resistance. Reports of reduced efficacy of antimalarial drugs due to resistance have emerged, particularly in Southeast Asia, raising alarms about the potential spread to Africa [43]. Similarly, there are concerns regarding the development of resistance to anthelmintic drugs used for soil-transmitted helminthiases. This situation underscores the urgent need for ongoing surveillance of drug efficacy and resistance patterns, as well as the development of new therapeutic strategies to combat parasitic infections effectively. To address these challenges, ongoing research is critical. There is a need for new drug discovery efforts focusing on novel targets within parasites and exploring combination therapies that may reduce the likelihood of resistance development. Additionally, public health initiatives aimed at improving diagnostic capabilities and access to treatment are essential for controlling these infections effectively. Furthermore, integrating community health education programs that promote preventive measures—such as improved sanitation for soil-transmitted helminths or vector control for malaria—can significantly reduce transmission rates and reliance on pharmacological treatments.

### 1.5. One-Health Approach

The One Health approach in West Africa addresses interconnected human, animal, and environmental health challenges, particularly zoonotic parasitic infections like cystic echinococcosis and cryptosporidiosis. These diseases disproportionately affect rural/peri-urban communities with high human–livestock interaction, such as farmers and abattoir workers. Collaborative efforts between veterinary and public health sectors integrate livestock vaccination, public education, and enhanced surveillance to curb transmission [25,44,45]. Climate-resilient strategies, including predictive modeling in Ghana, forecast malaria and schistosomiasis risks using climate data to guide proactive interventions. Despite persistent challenges—limited funding, fragmented coordination, and low public awareness—the growing adoption of One Health is driving policy reforms, capacity building, and investments to strengthen regional health security.

### 1.6. Public Health Challenges

Public health challenges related to parasitic infections in West Africa are significant, particularly in resource-poor settings where healthcare infrastructure is often inadequate. One of the primary challenges is the difficulty in diagnosing parasitic infections, which can be complicated by the nonspecific nature of symptoms and the lack of access to advanced diagnostic tools. Many healthcare facilities in rural areas may not have the capacity to perform laboratory tests necessary for accurate diagnosis, leading to underreporting and misdiagnosis [46]. Additionally, the reliance on clinical diagnosis without confirmatory testing can result in inappropriate treatment and increased transmission rates.

Surveillance systems for tracking parasitic infections are often limited in West Africa, hampered by insufficient funding, lack of trained personnel, and inadequate technological support. Many countries lack robust data collection mechanisms to monitor infection prevalence and treatment outcomes effectively. This deficiency complicates efforts to implement targeted interventions and allocate resources efficiently [47]. Moreover, disparities in accessing healthcare services further exacerbate the public health challenges posed by parasitic infections. Vulnerable populations, including rural communities and low-income families, often face barriers such as geographical distance to health facilities, high out-of-pocket costs for treatment, and a shortage of healthcare providers [48]. These disparities can lead to delayed diagnosis and treatment, increasing the risk of severe morbidity and mortality associated with parasitic infections. The combination of diagnostic challenges, limited surveillance capabilities, and inequitable access to healthcare underscores the urgent need for comprehensive public health strategies that address these systemic issues in West Africa.

The purpose of this systematic review is to provide a comprehensive analysis of parasitic infections in West Africa, focusing on their prevalence, effects on human health, public health challenges, current treatment options, and factors influencing transmission. By synthesizing the existing literature and research findings, this review aims to highlight the significant burden that parasitic diseases impose on affected populations and the healthcare systems in the region. Furthermore, it seeks to identify gaps in knowledge and areas requiring further investigation, particularly regarding the efficacy of current treatments and the emergence of drug resistance. Ultimately, this review aspires to inform public health strategies and interventions aimed at reducing the incidence and impact of parasitic infections in West Africa, thereby contributing to improved health outcomes and socio-economic development in the region.

## 2. Materials and Methods

A comprehensive search across various databases such as PubMed, Scopus, Web of Science, and Science Direct was conducted to find literature on parasites in West Africa. Using search terms such as key words related to “Parasites”, “Parasitic Infections”, “Treatments”, “Prevalence”, “Epidemiology”, and “West Africa”. In PubMed the Mesh Search Strategy was utilized for search terms such as “West Africa” and “Parasitic Infections” to enhance the search.

### 2.1. Study Selection

**Figure 1:**
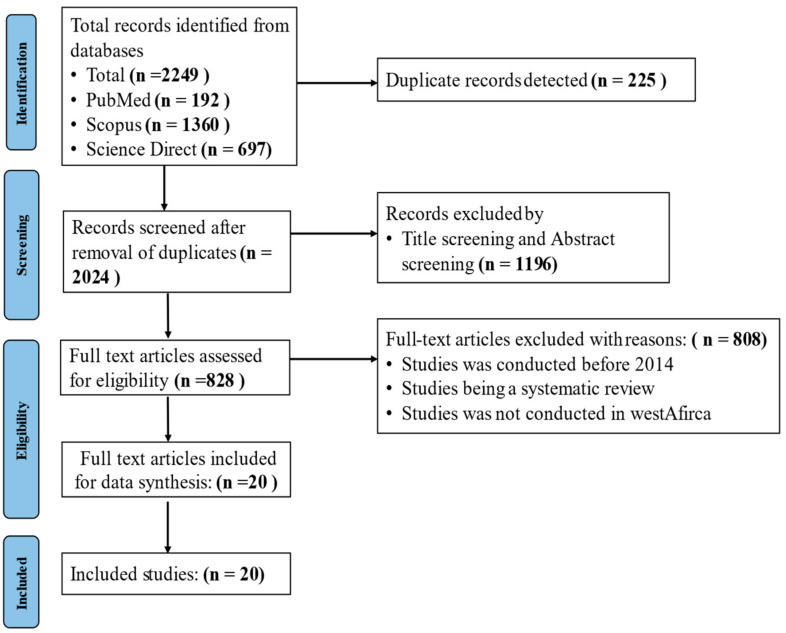
A flow chart showing the search process and the papers included in this study.

All publications were imported into the Rayyan online platform (Rayyan-Intelligent Systematic Review) and were screened for potential eligibility. Full-text articles were assessed for inclusion based on the predefined criteria (Figure 1). The search was limited to free, full studies published in English between 2014 and 2024 (Figure 1).

### 2.2. Inclusion Criteria

Publications included were cross-sectional and case–control studies reporting on prevalence, diagnostic techniques, and treatments being used to detect parasitic infections in any population.

### 2.3. Exclusion Criteria

Studies excluded were case reports, reviews, studies not conducted in West Africa, studies not published in English and studies with irrelevant information.

### 2.4. Data Extraction

Data extracted from the included papers were the author(s), year, country, region, study site, diagnostic techniques, treatments, target population, and the study period.

## 3. Results

Out of the 2249 papers identified from database, 808 were selected after screening and elimination of duplicates (Figure 1).

**Figure 2:**
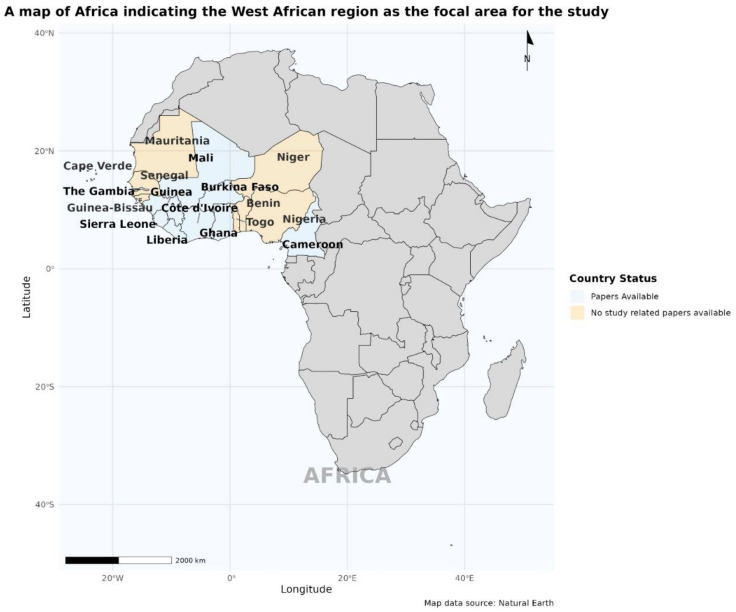
A map of Africa indicating the West African region as the focal area for this study.

The image below shows an African map indicating West Africa with the countries involved in this study. The countries with studies that qualified to be selected for this review are indicated in light/Columbia blue (Figure 2).

**Figure 3:**
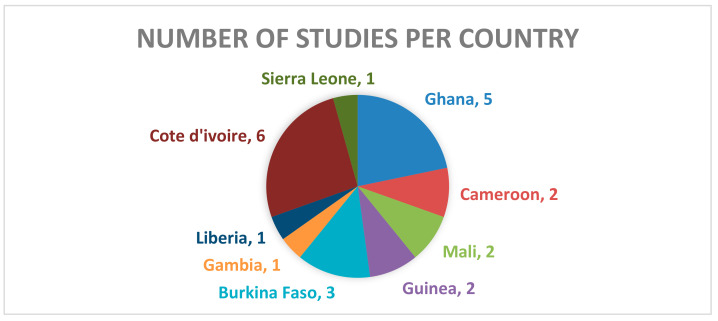
A pie chart showing the number of studies on treatment and diagnostic techniques in each country.

A few (7) West African countries had studies on diagnostics techniques and treatments with Cote d’ivoire and Ghana recording 6 and 5 respectively while Sierra Leone and Gambia recorded 1 each (Figure 3). 

**Figure 4:**
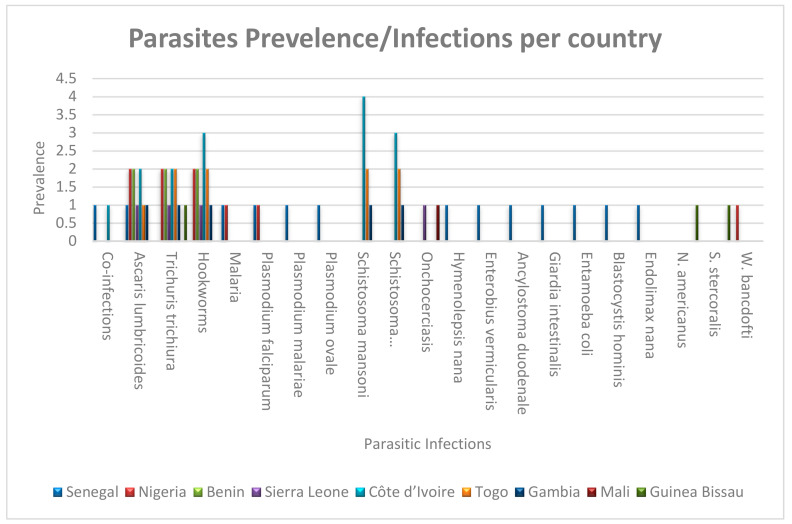
A bar graph depicting parasitic prevalence against infections recorded per country.

Various parasitic infections have been recorded across the different West African countries with different distribution patterns (Figure 4). The highest reported cases observed for *Plasmodium falciparum* and Onchocerciasis, with certain countries, such as Sierra Leone and Nigeria, showing notably higher incidences. Helminth infections, including *Ascaris lumbricoides*, *Trichuris trichiura*, and *Hookworms*, are also prevalent, with relatively consistent occurrences across multiple countries (Figure 4). Additionally, *Schistosoma haematobium* and *Schistosoma mansoni* infections are recorded, though at lower frequencies. Less common infections, such as *Hymenolepsis nana*, *Enterobius vermicularis*, and *Giardia intestinalis*, are reported in only a few instances, suggesting localized transmission patterns. The data indicate that while malaria remains a major concern, other parasitic infections contribute significantly to the disease burden in these regions, necessitating targeted public health interventions.

## 4. Discussion

Parasitic infections remain a formidable barrier to public health and socioeconomic development in West Africa. The region’s tropical climate, coupled with systemic challenges such as poverty, inadequate healthcare infrastructure, and environmental vulnerabilities, creates a fertile ground for the persistence of diseases like malaria, schistosomiasis, and soil-transmitted helminthiases (STHs). Despite decades of intervention, these diseases continue to disproportionately affect marginalized populations, underscoring the urgent need for innovative, context-specific strategies. This essay examines the complex dynamics of parasitic infections in West Africa, evaluates existing control measures, and advocates for integrated, sustainable solutions to achieve equitable health outcomes.

### 4.1. The Burden of Parasitic Diseases

West Africa’s parasitic landscape is dominated by malaria, a life-threatening disease transmitted by Anopheles mosquitoes. Endemic in rural and peri-urban areas, malaria accounts for significant morbidity and mortality, particularly among children under five and pregnant women. Similarly, schistosomiasis, transmitted through contact with freshwater contaminated by parasitic snails, plagues communities reliant on rivers and lakes for agriculture, domestic use, and recreation. Soil-transmitted helminths, including roundworms and hookworms, thrive in regions with poor sanitation, perpetuating cycles of malnutrition and anemia. Co-infections with multiple parasites are common, compounding the risk of chronic health complications. Neglected tropical diseases (NTDs) such as lymphatic filariasis and onchocerciasis, though targeted for elimination, persist in focal areas due to fragmented healthcare delivery and logistical challenges.

### 4.2. Drivers of Transmission: Ecology, Poverty, and Culture

The transmission of parasitic diseases in West Africa is deeply intertwined with environmental and socioeconomic factors. The region’s warm, humid climate and seasonal rainfall create ideal breeding conditions for vectors like mosquitoes and snails. Climate change exacerbates these risks, as rising temperatures and erratic weather patterns may expand the geographic range of parasites and their vectors. Socioeconomic disparities further fuel transmission: nearly 40% of West Africa’s population lives in poverty, limiting access to clean water, nutritious food, and healthcare. Urban slums and rural villages often lack adequate sanitation infrastructure, facilitating the spread of STHs. Cultural practices also play a role. For instance, farming and fishing communities are disproportionately exposed to schistosomiasis, while traditional beliefs about disease causation may delay treatment-seeking behavior. Gender inequities compound these risks, as women and girls often bear the burden of water collection, increasing their exposure to contaminated sources.

### 4.3. Progress and Pitfalls in Current Interventions

Significant strides have been made in combating parasitic diseases through mass drug administration (MDA), vector control, and public health campaigns. The African Programme for Onchocerciasis Control (APOC) and the Expanded Special Project for Elimination of Neglected Tropical Diseases (ESPEN) have reduced the prevalence of onchocerciasis and lymphatic filariasis by distributing ivermectin and albendazole to millions annually. Similarly, insecticide-treated bed nets and indoor residual spraying have contributed to a 20% decline in malaria mortality since 2000. However, these gains are fragile. Drug resistance, particularly partial resistance to artemisinin in malaria parasites and emerging ivermectin tolerance in some helminths, threatens to reverse progress. Mosquito resistance to pyrethroid insecticides and behavioral shifts, such as outdoor biting, also undermines vector control efforts. Sanitation campaigns, though critical for STH control, often fail due to insufficient funding and cultural resistance to latrine use.

### 4.4. Emerging Challenges in a Changing World

West Africa’s parasitic disease landscape is further complicated by contemporary crises. Rapid urbanization has led to overcrowded settlements with inadequate waste management, creating new hotspots for parasite transmission. Population displacement caused by conflict and climate disasters disrupts healthcare access, leaving displaced individuals vulnerable to outbreaks. Health systems, already strained by limited resources, face competing priorities such as HIV/AIDS and recurrent epidemics like Ebola and COVID-19. Meanwhile, climate change looms as an existential threat, altering disease patterns and overwhelming adaptive capacities.

### 4.5. Toward Sustainable and Equitable Solutions

Addressing West Africa’s parasitic disease burden demands a paradigm shift from siloed interventions to holistic, multisector approaches. A One Health framework, integrating human, animal, and environmental health, could mitigate zoonotic risks and enhance ecosystem resilience. Community engagement is equally vital: participatory health education programs can dispel myths about diseases, while local leaders can champion sanitation initiatives. Strengthening primary healthcare systems—through training community health workers and improving diagnostic capacity—will ensure early detection and treatment. Innovation must also be prioritized. Investment in research for novel therapies, such as malaria vaccines and broad-spectrum anthelminthic, is critical to overcoming resistance.

### 4.6. Recommendations

1. Longitudinal Studies: The need to court longitudinal studies as a means of tracking infection trends over time will provide deeper insights into the effectiveness of intervention strategies.
2. Impact Assessment: There is the need for research to be focused on assessing the impact of integrated control programs on both individual health outcomes and broader public health metrics.
3. Socioeconomic Factors: Investigating the influence of socioeconomic factors on infection prevalence can inform targeted interventions that address underlying vulnerabilities within communities.

## 5. Conclusions

The prevalence of malaria, schistosomiasis, and soil-transmitted helminths presents significant public health challenges in Sub-Saharan Africa. Addressing these issues through integrated control programs, targeted interventions, and ongoing research will be critical in reducing the burden of these diseases and improving health outcomes for vulnerable populations. This will also provide basis for the African region, most especially the West-African Sub-region to have a purpose and present in clearer terms, the dangers facing the continent in regard to eradicating some of these neglected and even not-so-neglected tropical diseases.
